# Superior vena cava-to-inferior vena cava bridging stent technique for cavo-atrial junction stenosis: a case series

**DOI:** 10.1093/ehjcr/ytag019

**Published:** 2026-01-30

**Authors:** Caoimhe Provost, Aziz Qazi, Sebastian Mafeld, Kongteng Tan, Graham Roche-Nagle, Cathal O’Leary

**Affiliations:** School of Medicine, University College Dublin, Belfield, Dublin 4, D04 C7X2, Ireland; Division of Vascular and Interventional Radiology, Department of Medical Imaging, University of Toronto, Toronto, ON, Canada M5T 1W7; Division of Vascular and Interventional Radiology, Department of Medical Imaging, University of Toronto, Toronto, ON, Canada M5T 1W7; Division of Vascular and Interventional Radiology, Department of Medical Imaging, University of Toronto, Toronto, ON, Canada M5T 1W7; Division of Vascular Surgery, Department of Surgery, University of Toronto, Toronto, ON, Canada M5T 1P5; Division of Vascular and Interventional Radiology, Department of Medical Imaging, University of Toronto, Toronto, ON, Canada M5T 1W7

**Keywords:** Bridging stents, Superior vena cava stenosis, Inferior vena cava stenosis, Cavo-atrial stenosis, SVC syndrome, Case series

## Abstract

**Background:**

Endovascular bridging stents are relatively underreported but effective methods to increase the diameter of stenosed segments of the superior and inferior cavo-atrial junction. This case report describes the use of superior to inferior vena cava bridging stents to resolve cavo-atrial junction stenoses in three patients with distinct mediastinal masses.

**Case Summary:**

All patients presented symptomatically with shortness of breath, as well as lower limb oedema, facial and neck swelling, and/or a cough. SVC-to-IVC bridging stents were selected as the appropriate intervention for these patients due to progressive symptoms, a lack of alternatives treatment options, and the anatomical proximity of the stenoses to the right atrium. More than 30-month follow-up showed a durable response in two patients without stent-related adverse events. One patient died 5 days post-procedure of shock of unclear aetiology.

**Discussion:**

In the two cases with long-term follow-up, patients’ symptoms have significantly improved, showing the effectiveness of this intervention for typically end-of-life patients. Consideration is given to the anatomical intricacies involved in the placement of stents, anticoagulation strategies, as well as the impact on future interventions.

**Conclusion:**

This series highlights the complexity of case management in patients with vena cavae stenosis, as well as the durability of bridging stent placement over a uniquely long follow-up.

Learning points

- SVC-to-IVC bridging stents offer a viable solution for complex central venous obstructions involving the cavo-atrial junction.
- The durability and safety profile demonstrated in two patients over 27 months of follow-up, highlights the potential of this technique for symptom relief in end-of-life patients.
- There is a need for careful patient selection, multidisciplinary planning, and thorough informed consent prior to bridging stent insertion in future patients.

## Introduction

Endovascular stenting provides a minimally invasive method for managing central venous obstruction to achieve effective early symptom relief.^1^ Cavo-atrial junctions stenoses are more complex due to mediastinal motion, limited stent landing zones, and proximity to critical cardiac structures. These challenges raise concerns about complications including cardiac tamponade or arrhythmias due to stents impinging on the myocardium.

The superior vena cava-to-inferior vena cava (SVC-to-IVC) bridging stent technique involves a stent that spans the obstructed segment and traverses the right atrium, effectively bridging the SVC to IVC. This approach helps with stent stability in anatomically complex cases when conventional techniques may not be feasible, and other therapeutic alternatives are not possible, e.g. chemotherapy or radiotherapy.^2^

**Figure:**
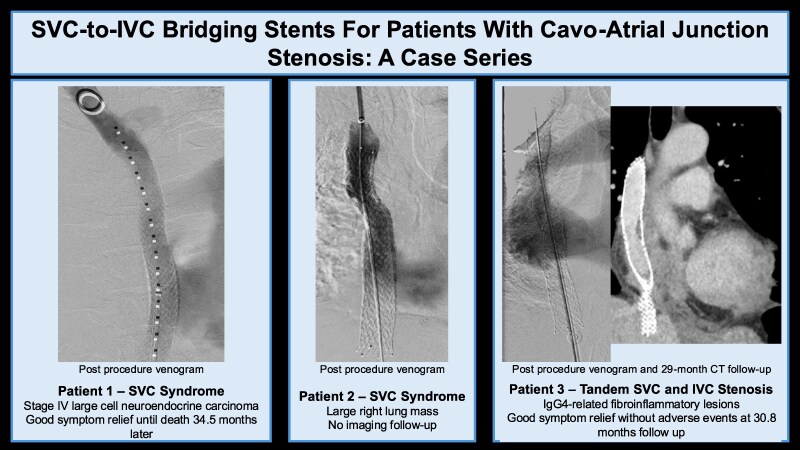


## Patient 1

**Figure 1:**
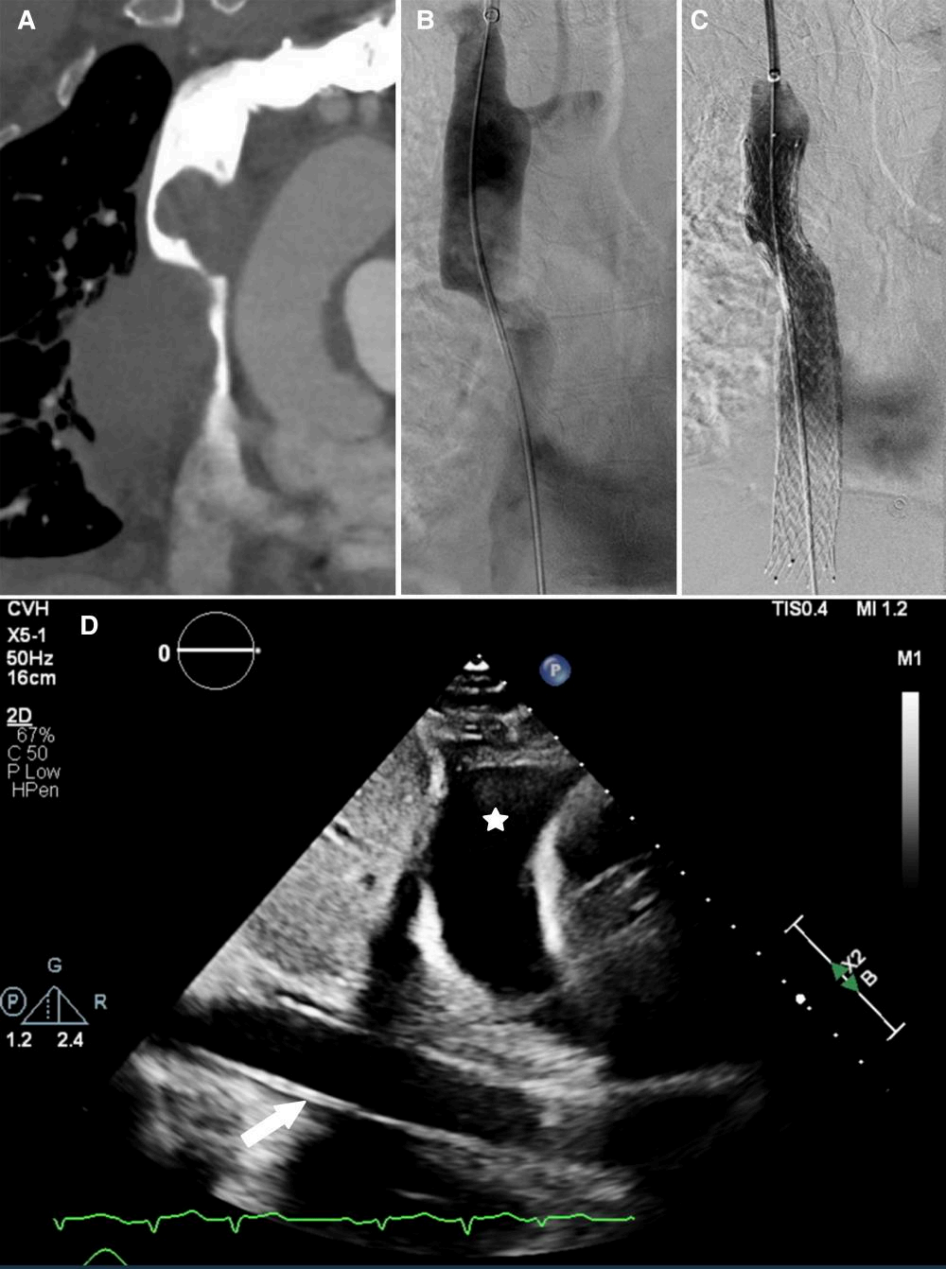
(*A*) Male in his 50 s (patient 1) with SVC syndrome related to squamous cell lung carcinoma. CT chest, coronal image, shows stenosis of the superior cavo-atrial junction due to the primary lung mass and mediastinal lymphadenopathy. (*B*) Superior vena cavagram shows severe stenosis of the superior cavo-atrial junction and upper right atrium due to extrinsic compression. (*C*) venogram post stent deployment shows relief of SVC stenosis with flow through the SVC-to-IVC bridging stent to the right atrium. (*D*) Transthoracic echocardiogram, subcostal view, identifies the IVC stent traversing the cavo-atrial junction (white arrow). A small pericardial effusion (white star) was unchanged from prior to the procedure and deemed too small to cause tamponade.

A male in his 50 s, with a history of treated pulmonary tuberculosis, presented to the ED with 3 weeks of progressive shortness of breath, cough, and tachycardia. On exam, there was plethora of his face and upper chest with a clear demarcation compared to his lower extremities. ECG showed narrow complex sinus tachycardia. Echocardiogram revealed normal ventricular systolic function and no valvular disease. A small pericardial effusion was present without findings of tamponade. CT chest showed a large right lung mass occupying the right hemithorax, with leftward tracheal deviation and severe narrowing of the superior cavo-atrial junction (*Figure 1A*). Cardiology consultation elicited a diagnosis of SVC syndrome based on his physical exam and CT findings.

The procedure was performed by an interventional radiologist, with local anaesthetic and moderate sedation with IV fentanyl and midazolam. An 8Fr sheath was inserted into the right common femoral vein. Venogram demonstrated near-complete occlusion of the upper SVC to the right atrium. No thrombus was present (*Figure 1B*). A 16 × 140 mm Zilver Vena stent (Cook Medical, Indiana, USA) was deployed from the upper SVC to the IVC. Serial balloon dilation was performed with 10 and 12 mm Mustang balloons (Boston Scientific Medical, USA) at the focal stenosis portion of the upper-mid bridging stent. Post-dilation venogram showed a moderate residual stenosis of the upper stent due to extrinsic compression. A 12 × 58 mm Lifestream stent graft (BD Medical, Canada) was deployed within the upper SVC stent to provide additional support. Venogram showed minimal residual stenosis (*Figure 1C*). Total fluoroscopy time was 8.8 min. Total contrast volume was 50 ml of iohexol. IV heparin was restarted due to previous intermittent atrial fibrillation.

The patient’s symptoms significantly improved. CT imaging the next day showed a patent IVC and SVC. Bronchoscopic biopsy confirmed primary squamous cell carcinoma of the lung. Five days post-procedure, and after return transfer to his local hospital, the patient developed shock of unclear aetiology. He remained in sinus tachycardia. Cardiology consultation and repeat transthoracic echocardiogram revealed RV diastolic collapse and pulsus paradoxus concerning for tamponade physiology (*Figure 1D*). His small pericardial effusion was unchanged and deemed insufficient to cause tamponade. His worsening condition was attributed to extracardiac tamponade from his large right lung mass. The patient’s shock progressed and they passed away 6 days post-procedure.

## Patient 2

**Figure 2:**
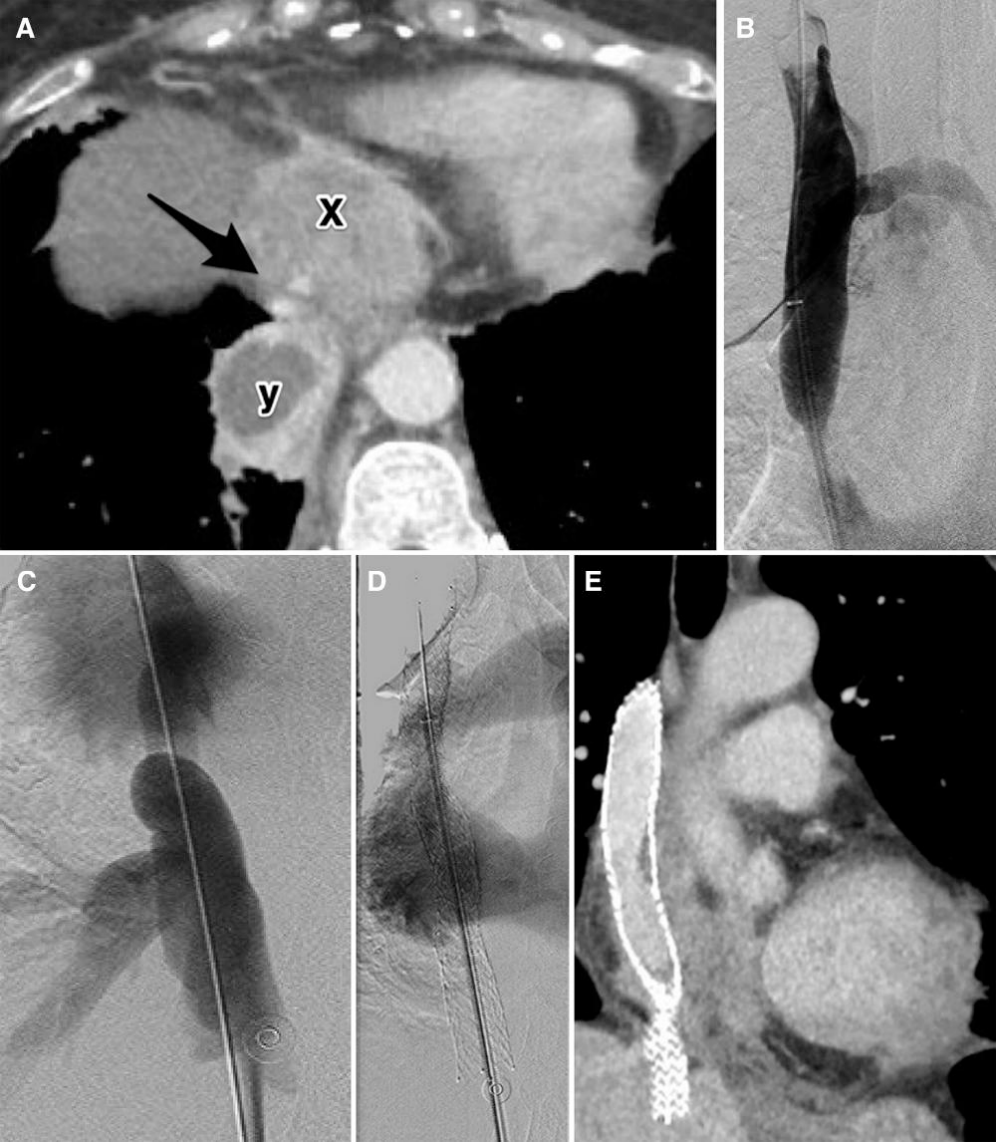
(*A*) Female in her 70 s presenting with IVC syndrome, ultimately diagnosed with IgG4 fibrosing disease (patient 2). **Axial image of a contrast-enhanced** CT chest shows a pericardial mass (x) causing severe stenosis of the suprahepatic IVC (arrow). Note is made of the known intrapulmonary sequestration (y). (*B*) Superior vena cavagram shows severe stenosis of the superior cavo-atrial junction due to extrinsic compression from a pericardial mass. (*C*) Inferior vena cavagram shows severe stenosis of the IVC above the confluence of the hepatic veins. The hepatic IVC is distended with reflux of contrast into dilated hepatic veins. (*D*) Venogram post stent deployment shows relief of SVC stenosis with flow through the SVC-to-IVC bridging stent to the right atrium. (*E*) CT chest 29 months post stent deployment shows a patent SVC-to-IVC bridging stent.

A female in her 60 s presented with several weeks of progressive bilateral lower extremity pitting oedema and shortness of breath at rest. She had a history of lumpectomy and left mastectomy for Phyllodes tumour 8 years prior and a history of chronic haemoptysis due to mycobacterium avium cellulare infection. On exam, she had prominent lower extremity oedema to her thighs with no upper extremity or facial oedema. CT imaging showed new pleural, pericardial, and myocardial masses. There was IVC stenosis due to two necrotic lesions in the pericardium and pleural space and SVC stenosis between mediastinal and pleural masses (*Figure 2A*). Transthoracic echocardiogram showed normal biventricular size and function, no pericardial effusion, and a non-dilated IVC that collapsed with inspiration. Cardiology consultation determined IVC compression as cause of her lower extremity oedema and advised IVC stent or radiotherapy to relieve IVC compression. She developed an increasing O2 requirement, increased lower extremity swelling, and hyperbilirubinemia while awaiting her radiation treatment. Ultrasound identified thrombus new hepatic vein thrombus. Intravenous heparin infusion was started and the decision made to proceed with stent placement in setting of IVC stenosis leading to hepatic vein thrombosis.

The procedure was performed in the interventional radiology suite with local anaesthetic and moderate sedation using intravenous midazolam and fentanyl. Right common femoral vein access was obtained and a 10Fr sheath inserted. Venogram showed severe stenoses of the superior and inferior cavo-atrial junction due to extrinsic compression, without thrombus (*Figure 2B* and *C*). A 16 × 140 mm Zilver Vena stent was deployed from the infra-azygous SVC into the supra-hepatic IVC. Post-deployment angioplasty was performed with a 14 mm Mustang balloon. Venogram showed no residual stenosis (*Figure 2D*). Total fluoroscopy time was 4.8 min. Total contrast volume was 50 mL of iohexol. Heparin infusion was restarted the next morning.

The patient’s symptoms improved significantly following the intervention. She was discharged 3 days later with low molecular weight heparin of 1.5 mg/kg q24h, dose reduced to 75% for IVC thrombus, malignancy, and history of haemoptysis. Haematology clinic follow up 1 month later changed to apixaban 5 mg PO twice daily. Repeat biopsy ultimately diagnosed a fibroinflammatory lesions consistent with IgG4-related disease. Follow-up CT imaging performed 29 months post-procedure demonstrated stent patency and unchanged position (*Figure 2E*). At most recent follow-up, 30.8 months after stent deployment, the patient reported mild bilateral lower extremity oedema, which is controlled with compression stockings. Of note, she developed atrial flutter 2 years post-stent placement, which is controlled on medical therapy. She was deemed not eligible for arrhythmia ablation due to the stent preventing access.

## Patient 3

**Figure 3:**
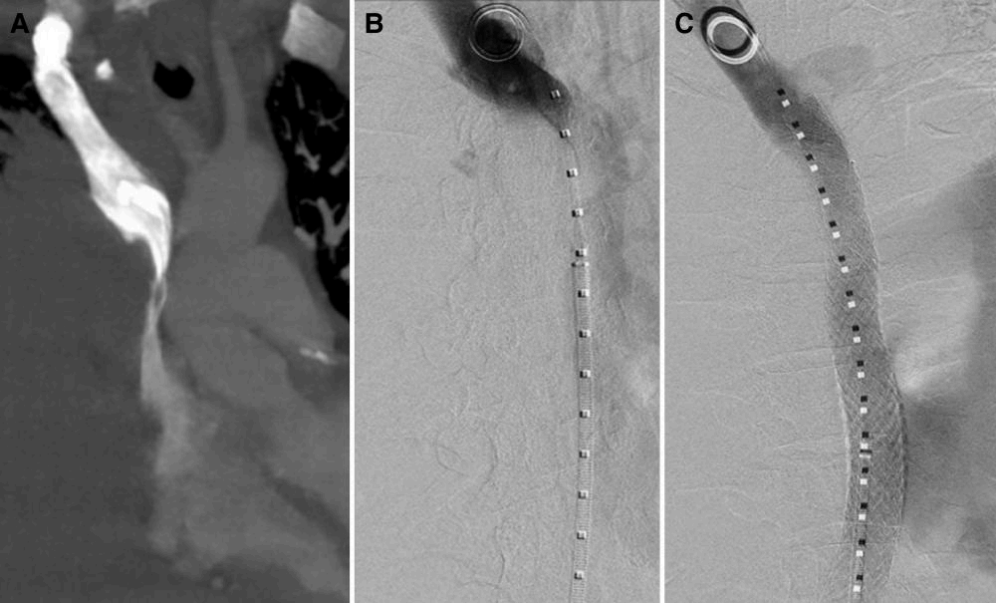
*(A)* A female in her 70s with SVC syndrome due to large cell neuroendocrine carcinoma of the lung (patient 3). CT chest, maximum intensity projection reconstruction in coronal plane, shows diffuse stenosis of SVC extending to superior cavo-atrial junction. *(B)* Venogram shows a pigtail catheter and sheath from a right femoral approach with pigtail in the right brachiocephalic vein. There is contrast filling the right brachiocephalic vein without central flow due to diffuse stenosis of the SVC extending to the superior cavo-atrial junction. *(C)* Venogram post stent deployment shows relief of SVC stenosis with flow through the SVC-to-IVC bridging stent to the right atrium.

A female in her 70 s, with no relevant past medical history, presented to the ED with 1 week of facial and neck swelling, dyspnoea, and intermittent chest pain. CT identified paratracheal and right mediastinal masses resulting in compression of the superior cavo-atrial junction and upper right atrium (*Figure 3A*). The diagnosis of SVC syndrome was made and an SVC-to-IVC bridging stent was planned due to the short landing zone.

The procedure was conducted by an interventional radiologist. Local anaesthesia alone was given as the patient was not fasting. Right jugular vein access was obtained provided and a 10-French sheath inserted. Venogram identified the severe superior cavo-atrial junction stenosis (*Figure 3B*). No thrombus was present. A 20 × 140 mm bare metal stent (Venovo, BD Medical, Canada) was deployed from the infra-azygous SVC to the subdiaphragmatic IVC. Post-deployment angioplasty was performed with a 16 mm Atlas Gold balloon (BD Medical, Canada). Venogram demonstrated a patent stent with brisk flow into the right atrium (*Figure 3C*). Total fluoroscopy time was 3.9 min. There were no immediate complications.

The patient’s symptoms significantly improved post-procedure. She was diagnosed with stage IV large cell neuroendocrine carcinoma of the lung. Upon discharge, she was prescribed therapeutic dose low molecular weight heparin (Tinzaparin). Her primary tumour was treated with radiotherapy and chemotherapy with carboplatin/etoposide.CT 25 months post stent placement confirmed stent patency and unchanged position. She passed away due to disease progression 34.5 months post procedure.

## Discussion

Endovascular stenting of the superior and/or inferior vena cava is an effective procedure that offers rapid symptom relief in a typically challenging clinical scenario. Stenting for extrinsic tumoral compression is typically reserved for palliation of symptoms, such as dyspnoea or pitting oedema, towards end-of-life, or emergent presentations where rapid decompression is the priority. A systematic review of IVC stent placement for malignant disease identified high technical success rates (100%) with a median primary patency of 78.5%. This was limited by a median follow-up of 1 month post stent placement range (1–2.9 months).^3^ Similar primary patency rates were reported in a review of SVC stenting for malignant SVC syndrome by Wong *et al*.^4^ Within the subset of patients with SVC and/or IVC obstruction, this case series describes the use of bridging stents to relieve superior cavo-atrial junction stenosis.

Cavo-atrial junction stenoses provide an anatomic challenge of anchoring a stent in a short landing zone. The primary concern is stent migration into the right atrium, which may lead to abnormalities in cardiac conduction, damage to the tricuspid valve, endocarditis, cardiac tamponade, and perforation.^2^ SVC-to-IVC bridging bare metal stent is proposed as a method of achieving stability while allowing venous return to the right atrium. This allows for the structural stability of the vessel, especially given the mobility of the right atrium throughout the cardiac cycle. Choice of stent diameter for the IVC or the SVC can be complicated by the fluctuation of vena cava diameter during respiration. To best adapt to this difficulty, some authors have advocated sizing the stent throughout a Valsalva manoeuvre and with a 20% margin for oversizing.^5^ Prior reports have described the successful use of SVC-to-IVC bridging stent in the palliative setting. These reports are limited by their short follow-up as all the patients passed away from their primary condition between 8 and 90 days post-procedure.^2,5–7^

Our case series describes two patients with >30-month follow-up without stent-related adverse events. To our knowledge, this represents the longest period of clinical follow-up post-bridging stent deployment in the current literature. The third patient passed away 6 days post-procedure of unclear aetiology. The patient had also undergone bronchoscopic biopsy in the days prior to his death. Stent-related pericardial tamponade or arrhythmia must be considered as a potential cause of death, although neither was noted as a cause of death on the clinical documentation. Long-term adverse events of stent thrombosis and/or occlusion are reported in up to 10.7% of SVC stents and 6–32% of IVC stents deployed for malignancy obstruction.^3,4^ The long-term stent patency for two patients in this case series may be due to the absence of tumour ingrowth from a primary malignancy and/or the long-term anticoagulation. Anticoagulation and/or antiplatelet therapy post central venous stent deployment is a complex decision guided by little evidence with wide differences in practice. Wong *et al*. found that the prescription of anticoagulation in patients with SVC stenting was largely mixed. Some clinicians initiated anticoagulation only when a SVC thrombosis was found, whereas others started therapy regardless of the presence of a thrombosis.^4^ Additionally, the specific anticoagulation agents prescribed is inconsistently reported in the literature, with evidence of warfarin, aspirin, heparin, and clopidogrel being used (for a unspecified duration).^4^ Both patients in this case series with long-term clinical follow-up continued on therapeutic anticoagulation with low-molecular weight heparin initially followed by oral factor-Xa inhibitor This may have contributed to long-term stent patency by preventing in-stent stenosis/thrombosis.

Patient selection for any central venous stent should prioritize individuals with symptomatic haemodynamic compromise, progressive obstruction, and limited alternative treatment options; in particular chemotherapy and radiotherapy. Cavo-atrial bridging stents are an option when the stenosis abuts the right atrium and prevents isolated SVC or IVC reconstruction. Stent placement is best undertaken when symptoms progress despite alternative therapies, but before deterioration to a level that increases procedural risk or precipitates haemodynamic instability.^^8^^ While traditionally used in palliative contexts, the long-term patency in this case series support the use of bridging stents when required for patients with benign aetiologies of cavo-atrial junction stenoses and a lack of alternative therapies.

One important point is the impact on future procedures, which has not previously been reported. One patient developed atrial flutter two years after stent placement. The stent has impacted her treatment options as she was deemed not eligible for arrhythmia ablation due to the stents precluding transvenous/transseptal access to her left atrium by the electrophysiology team. This is an important point to document in informed consent for patients undergoing bridging SVC-to-IVC stent placement.

## Conclusion

Endovascular SVC–IVC bridging stents are a viable option for symptomatic cavo-atrial junction stenosis in the absence of alternative therapies. In this series, two patients demonstrated durable patency beyond 30 months with sustained symptomatic improvement and no stent-related complications. These examples underscore the technical complexity and potential long-term efficacy of bridging stent reconstruction in advanced vena cava obstruction.

## Lead author biography


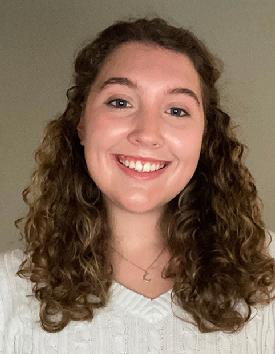


Caoimhe Provost is a medical student at the University College Dublin.

## Supplementary Material

ytag019_Supplementary_Data

## Data Availability

All data underlying this case series is included within the article. Additional details are available from the corresponding author upon reasonable request.
