# Supplementary material for: Superior vena cava-to-inferior vena cava bridging stent technique for cavo-atrial junction stenosis: a case series
Source: Eur Heart J Case Rep. 2026 Jan 30;10(2):ytag019. doi: 10.1093/ehjcr/ytag019 (PMC12908081; doi:10.1093/ehjcr/ytag019)
Supplement: ytag019_Supplementary_Data [file ytag019_supplementary_data.doc]

**Lack of written consent form**

This form should be used by authors wishing to submit a case report/case series/grand round/images in cardiology article to *European Heart Journal – Case Reports* where written consent is not available for a patient included in the manuscript.

The *Editorial Board* of *European Heart Journal – Case Reports* believes that patients should consent to the publication of their cases. However, it is also appreciated that there are some circumstances where the ability to gain informed consent is not possible or appropriate.

Please use the lack of written consent flow chart to confirm the action that is required. This form should only be used as outlined in this flowchart.

**Manuscript Title:** ………………………SVC-to-IVC Stent Technique for Cavo-Atrial Junction Stenosis of the Superior and Inferior Vena Cava: Case Series…………………

**Manuscript ID (if known):** ………………………………………NA………………………………………………

**Scenario A – Witnessed verbal consent to publish has been obtained from the patient but written consent is not possible**

*A.1. Please outline the reason(s) why written consent from the patient has not been possible in this situation:*

……………………………………………………………………………………………………………………………………………………………

……………………………………………………………………………………………………………………………………………………………

……………………………………………………………………………………………………………………………………………………………

*A.2 Who witnessed the verbal consent*……………………………………………………………………………………………..

…………………………………………………………………………………………………………………………………………………………..

A.3 *If possible, please provide the date of the witnessed consent*……………………………………………………………………………………………………………………………………………….

…………………………………………………………………………………………………………………………………………………………………………………………………………………………………………………………………………………………….

**Scenario B – Verbal consent to publish has been obtained from the patient but not witnessed and written consent is not possible**

*B.1. Please outline the reason(s) why written consent from the patient has not been possible in this situation:*

……………………………………………………………………………………………………………………………………………………………

……………………………………………………………………………………………………………………………………………………………

……………………………………………………………………………………………………………………………………………………………

*B.2. Please outline the reason(s) why witnessed verbal consent from the patient has not been possible in this situation:*

……………………………………………………………………………………………………………………………………………………………

……………………………………………………………………………………………………………………………………………………………

……………………………………………………………………………………………………………………………………………………………

**Scenario C – The patient is deceased and while there are next-of-kin/surviving relatives, it is not possible to contact them.**

*C.1. Please outline the reason(s) why the next-of-kin/surviving relative(s) cannot be contacted in this situation:*

………………………The only available contact details in the electronic medical record are out of date. ………………………………………

……………………………………………………………………………………………………………………………………………………………

……………………………………………………………………………………………………………………………………………………………

*C.2. If the next-of-kin/surviving relatives contact details are not available, please outline what attempts have been made to obtain these details:*

…………………………4 telephone calls, 1 week apart, to try to contact patients. Email sent to each available email address. …………………………………………

……………………………………………………………………………………………………………………………………………………………

……………………………………………………………………………………………………………………………………………………………

**Scenario D – The patient is deceased and has no surviving relatives / next-of-kin.**

*D.1. Please provide details of this scenario and how it has been confirmed that there are no appropriate surviving relatives to contact:*

……………………………………………………………………………………………………………………………………………………………

……………………………………………………………………………………………………………………………………………………………

……………………………………………………………………………………………………………………………………………………………

**Scenario E – The patient is alive but has not been contacted to seek consent to publish due to legal or ethical restrictions.**

*E.1. Please provide details of the legal and/or ethical restrictions that prevent contact with the patient to seek consent to publish:*

……………………………………………………………………………………………………………………………………………………………

……………………………………………………………………………………………………………………………………………………………

……………………………………………………………………………………………………………………………………………………………

**Scenario F – The patient is alive but has not been contacted to seek consent to publish and there are no contact details for the patient.**

*F.1. Please provide details of the steps taken by the authors to obtain contact details for the patient:*

……………………………………………………………………………………………………………………………………………………………

……………………………………………………………………………………………………………………………………………………………

……………………………………………………………………………………………………………………………………………………………
